# Tuberculosis-related hospitalizations in Brazil: a nine-year experience in a high-burden setting

**DOI:** 10.1016/j.bjid.2026.105789

**Published:** 2026-02-21

**Authors:** Gabriela Pizarro Ossa Ferro Henriques, Guilherme Barbosa Pinto, Erika Yukie Ishigaki, Nicoly Caroline de Andrade Delmondes, Daniel Ayabe Ninomiya, Olavo Henrique Munhoz Leite, Marcello Mihailenko Chaves Magri

**Affiliations:** a Centro Universitário Faculdade de Medicina do ABC (FMABC), Disciplina de Infectologia, Santo André, SP, Brazil; b Hospital das Clínicas da Faculdade de Medicina da Universidade de São Paulo (HCFMUSP), Divisião de Clínica de Moléstias Infecciosas e Parasitárias, São Paulo, SP, Brazil

**Keywords:** Tuberculosis, Hospitalization, TB-HIV coinfection, Risk factors

## Abstract

**Background:**

Tuberculosis (TB) remains a major public health concern globally and in Brazil. Although ambulatory treatment is recommended for most patients, hospitalizations still occur due to severe clinical presentations, social vulnerability, or treatment complications.

**Methods:**

We conducted a retrospective, observational study of TB-related hospitalizations at a regional referral tertiary public hospital in São Paulo, Brazil, from 2013 to 2022. Medical records were reviewed to collect epidemiological, clinical, laboratory, and microbiological data.

**Results:**

Among 197 hospitalizations for TB, 73.1% were male, with a mean age of 41.8 years. TB-HIV coinfection was identified in 55.3% of cases, with 83.5% of these patients having CD4+ T-cell counts ≤ 200 cells/mm^3^. Malnutrition (40.1%), smoking (64.0%), alcohol use (51.6%), and illicit drug use (58.1%) were common. Pulmonary TB occurred in 48.7%, while 29.4% had extrapulmonary TB and 21.8% disseminated forms. Diagnostic confirmation was achieved in 74.1%, including bacilloscopy, culture, molecular test, ADA and biopsy. The main reason for hospitalization was diagnostic work-up (74.1%), with a median symptom duration of 3.6 months. ICU care was required in 23.8% of cases. In-hospital mortality was 17.3%, and significant risk factors included malnutrition and thrombocytopenia.

**Conclusions:**

In this high-burden setting, TB hospitalizations were associated with social vulnerability, HIV coinfection, and delayed diagnosis. The high frequency of severe presentations highlights the importance of early detection and access to molecular testing. Improved integration between outpatient care and hospital services may reduce the need for hospitalization and improve outcomes.

## Introduction

Tuberculosis (TB) remains a leading cause of morbidity and mortality worldwide, with more than 7.5 million new cases and 1.3 million deaths annually^1^, ^2^, ^3^, ^4^ Brazil is among the 30 high-burden countries, reporting 84,308 new cases in 2024 and 6,025 deaths in 2023^1^, ^2^, ^3^ Pulmonary TB accounts for 80%–85% of cases and drives transmission, while extrapulmonary forms are more frequent in immunocompromised individuals, particularly those living with HIV/AIDS (PLWHA)^1^^,^^3^^,^^5^, ^6^, ^7^, ^8^

Although treatment under the Unified Health System (SUS) is predominantly ambulatory, hospitalizations remain necessary in selected situations, including severe disease, drug toxicity, treatment nonadherence, and social vulnerability^3^^,^^4^^,^^9^, ^10^, ^11^, ^12^ Between 2011 and 2020, Brazil recorded more than 140,000 TB-related hospitalizations and 11,672 in-hospital deaths, mostly in young adults^2^^,^^3^^,^^13^ HIV infection is the main comorbidity associated with TB admissions and mortality,^2^^,^^3^^,^^5^^,^^14^ with 9,624 new TB-HIV coinfections reported in Brazil in 2024^2^ Other risk factors include malnutrition, diabetes, alcohol use, smoking, and marginalized social groups such as people deprived of liberty, individuals experiencing homelessness, transgender people, and Indigenous populations^3^^,^^7^^,^^15^, ^16^, ^17^, ^18^^,^^19^

In this context, evaluating TB-related hospitalizations can provide critical insights into diagnostic gaps, social vulnerabilities, and clinical risk factors for poor outcomes. This study describes tuberculosis-related hospitalizations over a ten-year period in a tertiary hospital in São Paulo, Brazil, with emphasis on reasons for admission and predictors of in-hospital mortality.

## Methods

We conducted a retrospective, observational descriptive study of hospitalizations for TB at Hospital Estadual Mario Covas (HEMC), a tertiary public teaching hospital located in Santo André, São Paulo, Brazil. The hospital provides high-complexity care and is the largest referral facility in the Greater ABC metropolitan region, covering seven municipalities and operating under the regulation of the state system. The study period covered January 2013 through December 2022. This study was approved by the Institutional Review Board of the Centro Universitario FMABC (CAAE: 69610323.4.0000.0082) and authorized by the HEMC clinical directorate. All data were obtained from electronic medical records and were de-identified prior to analysis.

Eligible cases included adult patients (≥18-years) hospitalized with a diagnosis of TB at admission or diagnosed during hospitalization, identified either in the electronic medical records or through notification in the national reporting system (SINAN). Patients were excluded if medical records were unavailable, if the diagnosis of TB was ruled out during hospitalization, or if they had been previously diagnosed and receiving treatment for more than 30-days when hospitalization was unrelated to TB. Cases were classified as pulmonary when only the lungs were affected, extrapulmonary when a single site outside the lungs was involved, and disseminated when two or more non-contiguous sites were affected or when there was bone marrow involvement or a positive blood culture. Information collected included site of admission (ward or ICU), length of stay, need for intensive care, treatment modality (empirical or microbiologically confirmed), outcomes such as discharge, death, transfer, or discharge against medical advice, and TB-related readmissions.

Medical records were reviewed through progress notes, discharge summaries, and prescription logs. Patients were included if anti-TB therapy was initiated during hospitalization or if discharge diagnosis included TB-related codes. Data collected included age, sex, race/ethnicity, education level, and place of residence. Behavioral risk factors (smoking, alcohol use, illicit drug use), history of incarceration or homelessness, and known contact with TB cases were recorded. Comorbidities included HIV infection, Diabetes Mellitus (DM2), Chronic Kidney Disease (CKD), chronic liver disease, malignancy, and use of immunosuppressive therapies. Comorbidities were not considered mutually exclusive and were recorded based on clinical documentation at admission and during hospitalization. When applicable, overlapping conditions were reported accordingly.

HIV status was classified as known prior to admission or diagnosed during hospitalization. For people living with HIV/AIDS (PLWHA), data on CD4+ T-cell count, and HIV Viral Load (VL) were collected within three months before or after admission. Nutritional status was assessed by the hospital nutrition team using validated criteria based on Body Mass Index (BMI) and arm circumference, according to WHO (1995), PAHO (2001), Frisancho (1990), and Barbosa (2005) standards. TB history and treatment initiation date were retrieved from the notification platform of the São Paulo State Health Department.

Diagnostic tests reviewed included Acid-Fast Bacilli (AFB) smears, *M. tuberculosis* cultures, conventional and rapid PCR assays, histopathology of tissue biopsies, and ADA levels in cavitary fluids. Specimens included sputum, tracheal aspirates, Bronchoalveolar Lavage (BAL), Cerebrospinal Fluid (CSF), pleural, pericardial, peritoneal, synovial fluids, urine, lymph node aspirates, and blood cultures. Positive results were defined as follows: AFB smear: any positive result regardless of specimen, detection of *M. tuberculosis* DNA, “trace detected” results were accepted in PLWHA or suspected extrapulmonary TB, identification of *M. tuberculosis* complex, or non-specified “positive” cultures when treatment was initiated by the attending physician, granulomatous inflammation with or without caseous necrosis, ADA: > 40 U/L in pleural fluid; > 30 U/L in other fluids, or > 9 U/L in CSF, when interpreted as TB by the clinical team, and results from external labs were included if documented in the chart and considered in clinical decision-making. We recorded hemoglobin, total leukocyte and platelet counts, C-Reactive Protein (CRP), and serum albumin at admission (within 5 days), prioritizing the earliest result.

Hospitalizations were grouped as: Pre-established TB diagnosis, hospitalization due to drug-related adverse events (e.g., hepatotoxicity, skin reactions), respiratory worsening, clinical deterioration from treatment discontinuation, or persistent TB-related symptoms; Diagnostic work-up: admission for investigation of unexplained general symptoms, respiratory failure, neurological signs, or abscess drainage.

Descriptive statistics included frequencies, percentages, means with Standard Deviation (SD), medians, and ranges. Categorical variables were compared using the Chi-Square test or Fisher’s exact test, as appropriate. Adjusted standardized residuals were used to explore differences in distribution. Associations between clinical variables and in-hospital mortality were explored. All statistical analyses were performed using IBM SPSS Statistics (version 20.0; IBM Corp., Armonk, NY, USA). A two-sided p-value < 0.05 was considered statistically significant.

## Results

**Fig. 1:**
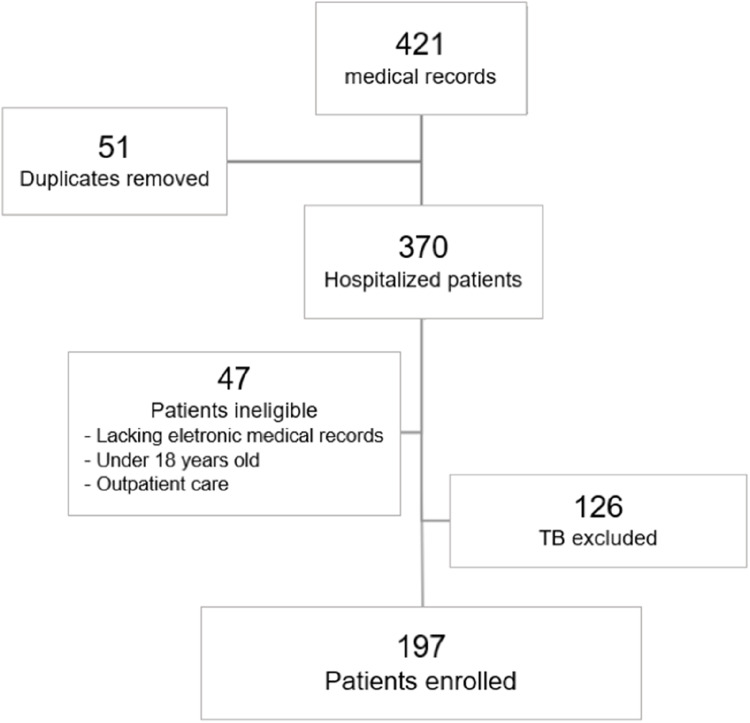
Flow diagram of medical record screening and patient inclusion.

Of 421 medical records reviewed, 197 TB-related hospitalizations were included in the final analysis. Exclusions and the selection process are detailed in the inclusion flowchart (Fig. 1). Among the 197 patients, 144 (73.1%) were male, and the mean age was 41.8 years (SD = 13.8). Regarding self-reported race, 109 (55.3%) identified as mixed-race (pardo), 78 (39.6%) as white, and 10 (5.1%) as Black. Most patients resided in the Greater ABC region (87.8%), primarily from Santo André (38.3%). Transgender women accounted for 2.5% of hospitalizations. Among the 113 patients with available education data, 60.2% had not completed primary school. Behavioral and social vulnerabilities were common. Among the 128 patients with available data, 64.0% reported tobacco use, 51.6% alcohol consumption, and 58.1% illicit drug use. Malnutrition was documented in 79 patients (40.1%). A total of 36 patients (18.2%) had no documented comorbidities. The characteristics of patients hospitalized with tuberculosis are presented in Table 1.

**Table 1:** Characteristics of 197 Patients Hospitalized for TB from 2013 to 2022.

Characteristics	n/n (%) or Mean (±SD)
**Age (years)**	41.8 (±13.8)
**Gender**	
Male	144/197 (73.1%)
Female	48/197 (24.4%)
Transgender Women	5/197 (2.5%)
**Race/Color**	
Mixed race	109/197 (55.3%)
White	78/197 (39.6%)
Black	10/197 (5.1%)
**Educational Level**	
No formal education	3/113 (2.6%)
Elementary school (1 to 9 yrs)	68/113 (60.2%)
High school (10 to 12 yrs)	40/113 (35.4%)
Higher education (> 12 yrs)	2/113 (1.8%)
**Habits**	
Smoking	82/128 (64.0%)
Alcoholism	66/128 (51.6%)
Other Illicit Drug Use	68/117 (58.1%)
**Comorbidities (Mutually Exclusive Groups)**	
No comorbidities	36/197 (18.2%)
HIV infection only (PLHIV)	53/197 (26.9%)
PLHIV + other comorbidities	8/197 (4.1%)
Malnutrition only	31/197 (15.7%)
PLHIV + Malnutrition	48/197 (24.4%)
**Specific Comorbidities (Overlapping Findings)**^a^	
Type 2 Diabetes (DM2)^a^	13/197 (6.6%)
Chronic liver disease^a^	10/197 (5.1%)
Chronic kidney disease^a^	6/197 (3.0%)
Other comorbidities^a^	11/197 (5.6%)
**Patients with TB and HIV**	
Concurrent HIV diagnosis	35/109 (32.1%)
- Detectable Viral Load (VL)	33/35 (94.3%)
- CD4 < 200 cells/mm^3^	29/35 (82.9%)
- CD4 > 200 cells/mm^3^	4/35 (11.4%)
Previous HIV diagnosis	74/109 (67.9%)
- Detectable Viral Load (VL)	58/74 (78.4%)
- CD4 < 200 cells/mm^3^	62/74 (83.8%)
- CD4 > 200 cells/mm^3^	11/74 (14.9%)
Deaths in TB-HIV subgroup	22/109 (20.2%)
- Deaths TB-HIV-Related Malnutrition	13/22 (59.1%)
**Clinical Presentation**	
Pulmonary TB	96/197 (48.7%)
Extrapulmonary TB	58/197 (29.4%)
- Central Nervous System (CNS)	29/58 (50.0%)
- Pleural	13/58 (22.4%)
- Lymph Node	4/58 (6.9%)
- Osteoarticular	4/58 (6.9%)
- Other (peritoneum, pericardium, etc.)	8/58 (13.8%)
Disseminated TB	43/197 (21.8%)
- Bone Marrow Culture	1/43 (2.3%)
- 2 Locations	36/43 (83.7%)
- 3 Locations	4/43 (9.3%)
- 4 Locations	2/43 (4.6%)
**Reason for Hospitalization**	
**Established TB Diagnosis**	**51/197 (25.9%)**
- Adverse Drug Reaction	14/51 (27.4%)
— Hepatotoxicity	11/14 (78.6%)
— Medication Intolerance	2/14 (14.3%)
— Drug Eruption	1/14 (7.1%)
- Respiratory Deterioration	9/51 (17.7%)
- Treatment Interruption / Symptom Deterioration	10/51 (19.6%)
- Neurological Symptoms	9/51 (17.7%)
- General Symptoms (fever, weight loss)	5/51 (9.8%)
- Other Reasons	4/51 (7.8%)
**Diagnostic Elucidation**	**146/197 (74.1%)**
- Respiratory Insufficiency	43/146 (29.5%)
- Neurological Symptoms	41/146 (28.1%)
**Outcomes & Management**	
Empirical Treatment	51/197 (25.9%)
Total In-hospital Deaths	34/197 (17.3%)

SD, Standard Deviation; TB, Tuberculosis; HIV, Human Immunodeficiency Virus; PLHIV, People Living with HIV.

a Comorbidity groups are mutually exclusive for statistical distribution, while specific conditions (DM2, Liver/Kidney disease) are reported as overlapping clinical findings. Respiratory deterioration indicates clinical worsening, whereas respiratory insufficiency refers to acute respiratory failure leading to admission.

HIV coinfection was diagnosed in 109 patients (55.3%). Among them, 83.5% had CD4+ T-cell counts ≤ 200 cells/mm^3^, and other 83.5% had detectable HIV viral loads at admission. The median CD4 count was 57 cells/mm^3^ (range: 1–712). The in-hospital mortality rate among HIV-positive patients was 20.2% (22/109), and among those who died, 59.1% were also malnourished. Diabetes mellitus was present in 13 patients (6.6%), chronic liver disease in 10 (5.1%), and chronic kidney disease in 6 (3.0%). Four patients had other immunosuppressive conditions, including hematologic malignancy or use of corticosteroids or biologics.

Pulmonary TB accounted for 48.7% of hospitalizations, extrapulmonary TB for 29.4%, and disseminated forms for 21.8%. Among extrapulmonary cases, the most commonly affected site was the central nervous system (50.0%), followed by pleura (22.4%), lymph nodes (6.9%), and bones or joints (6.9%). Less frequent sites included the pericardium, peritoneum, psoas muscle, and intestine. Among patients with disseminated TB, 83.7% had two or more noncontiguous sites involved, 9.3% had three sites, and 4.6% had four sites. One patient had isolated bone marrow involvement with positive cultures but no other identified focus.

The leading reason for hospitalization was diagnostic investigation (74.1%), primarily due to respiratory failure (29.5%), neurological symptoms (28.1%), or nonspecific systemic complaints (34.2%). In the remaining patients, TB had been previously diagnosed and admission was related to adverse drug reactions (27.4%), clinical worsening following treatment interruption (19.6%), respiratory deterioration (17.7%), or neurologic complications (17.7%).

Most patients (90.4%) were initially admitted to general medical wards. Intensive care was required in 47 cases (23.8%), with a median ICU stay of 14-days (range: 1–97). Median total hospital stay was 22-days (range: 1–277). A total of 152 patients (77.2%) were discharged alive, 34 (17.3%) died during hospitalization, 4 left against medical advice, and 7 were transferred. There were 24 TB-related readmissions, most of which were due to clinical deterioration (75%).

The median time from symptom onset to hospital admission among patients admitted for diagnostic work-up was 3.6 months. The interval from admission to initiation of anti-TB therapy ranged from 0 to 51 days, with a mean of 9-days. 74.1% of patients had Tuberculosis diagnostic confirmation, including microbiology, molecular biology, biopsy and ADA test. Diagnostic test performance varied significantly by sample type (Table 2). Respiratory specimens yielded the highest positivity rates across all modalities: 51.8% for smear microscopy, 32.2% for culture and 67.5% for molecular assays. In contrast, yields were considerably lower in Cerebrospinal Fluid (CSF), with 2.3% positivity for smear, 2.0% for culture, and 24.1% for molecular testing. Serous fluids showed no smear positivity and was low for culture (4.5%) as well, with molecular positivity of 33.3% in the few tested cases, the ADA was positive in 10%. Among lymph node, urine, and other samples, molecular testing was the most effective (66.6%), followed by culture (16.7%). 40% of all biopsies were interpreted as suggestive of tuberculosis. These findings underscore the diagnostic challenges of extrapulmonary TB and the utility of combining diagnostic strategies. A total of 25.9% of patients were treated empirically without bacteriological confirmation.

**Table 2:** Positivity rates of smear microscopy, culture, and molecular testing by sample type (n = 197).

Sample Type (n)	Test Type	Positive / Total (%)
Respiratory Samples (323)	Smear microscopy	87/168 (51.8%)
	Culture	37/115 (32.2%)
	Xpert + PCR	27/40 (67.5%)
CNS (121)	Smear microscopy	1/43 (2.3%)
	Culture	1/49 (2.0%)
	Xpert + PCR	7/29 (24.1%)
Serous Fluids (45)	Smear microscopy	0/17 (0%)
	Culture	1/22 (4.5%)
	Xpert + PCR	2/6 (33.3%)
Lymph Nodes + Urine + Others (76)	Smear microscopy	3/13 (23.1%)
	Culture	9/54 (16.7%)
	Xpert + PCR	6/9 (66.6%)
Total (n = 565)	Smear microscopy	91/241 (37.8%)
	Culture	48/240 (20%)
	Xpert + PCR	45/84 (53.6%)

CNS, Central Nervous System; PCR, Polymerase Chain Reaction; Xpert, Xpert MTB/RIF assay; Smear microscopy, Ziehl-Neelsen or auramine staining for acid-fast bacilli; For “Total”, the denominator corresponds to the number of samples tested with each respective method, not the overall number of samples.

When comparing survivors and non-survivors (Table 3), malnutrition was significantly more prevalent among patients who died (61.8% vs. 38.7%; p = 0.011), as was thrombocytopenia (32.4% vs. 15.3%; p = 0.022). Although HIV infection, CD4+ counts ≤200 cells/mm^3^, and detectable viral load were more common among non-survivors, these associations did not reach statistical significance. A trend toward higher mortality was also noted among patients with anemia (82.4% vs. 68.7%; p = 0.079) and hypoalbuminemia (mean 2.4 vs. 2.79 g/dL; p = 0.133). No significant differences were found with respect to TB form (pulmonary, extrapulmonary, or disseminated), leukocytosis, or elevated C-reactive protein.

**Table 3:** Characteristics of 197 TB patients according to survival outcome during hospital admission.

Epidemiological characteristics	Survival (n = 163)	Death (n = 34)	p^c^
Age (years)^b^	41.18 ± 13.49	44.76 ±15.27	0.374
Male	122 (74.8)	27 (79.4)	0.374
Smoking	70 (65.4)^c^	12 (57.1)^c^	0.314
Alcoholism	57 (52.8)^c^	9 (45.0)^c^	0.346
Illicit drugs	61 (59.8)^d^	6 (42.9)^d^	0.180
No comorbidities	51 (31.3)	5 (14.7)	0.079
HIV infection	87 (53.4)	22 (64.7)	0.154
CV-HIV detectable	74 (85.1)	17 (77.3)	0.571
LTCD4 ≤ 200 cels/mm^3^	70 (80.5)	19 (86.4)	0.260
Immunosuppressive comorbidities	20 (12.3)	7 (20.6)	0.156
Malnutrition	63 (38.7)	21 (61.8)	0.011
Clinical features			
Pulmonary TB alone	82 (50.3)	14 (41.2)	0.218
Extrapulmonary TB	47 (28.8)	10 (29.4)	0.548
Disseminated TB	34 (20.9)	10 (29.4)	0.192
Laboratory characteristics			
Hb ≤ 12 g/dL	112 (68.7)	28 (82.4)	0.079
Leukocytes ≥ 10,000 cells/mm^3^	53 (32.5)	13 (38.2)	0.325
Plaquetas < 150.000 plaquetas/mm^3^	25 (15.3)	11 (32.4)	0.022
C-reactive protein^b^	10.66 ± 9.38	12.76 ± 8.81	0.433
Albumin^b^ (g/dL)	2.79 ± 0.57	2.4 ± 0.50	0.133

PR, Prevalence Ratio; CI, Confidence Interval; SD, Standard Deviation; HIV, Human Immunodeficiency Virus; CV-HIV, HIV Viral load; LTCD4, CD4 T-Lymphocytes; TB, Tuberculosis; Hb, Hemoglobin.

a Values expressed in n (%), except where indicated.

b Values expressed as mean ± standard deviation.

c Information contained in 128 medical records.

d Information contained in 117 medical records.

5 Immunosuppressive comorbidities include type 2 diabetes mellitus, chronic kidney disease, chronic liver disease, oncohematologic neoplasms, use of immunobiologicals, and use of corticosteroids in immunosuppressive doses

**Fig. 2:**
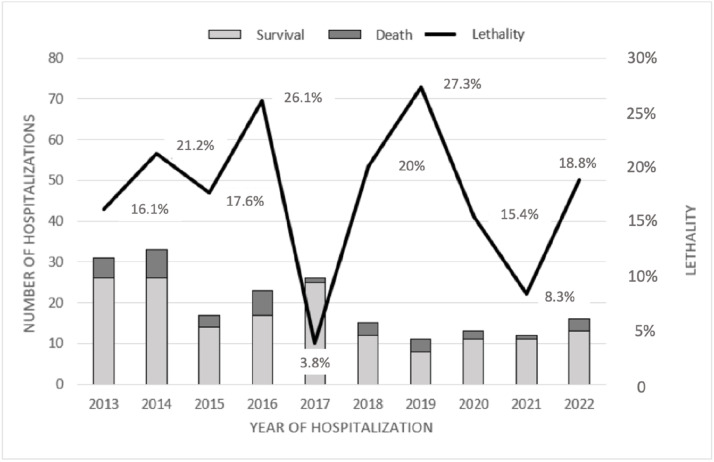
Hospital lethality in patient admitted with tuberculosis.

The in-hospital mortality rate remained relatively stable across the ten-year study period, with no abrupt increases during the COVID-19 pandemic years (2020–2021). As shown in Fig. 2, the year-to-year lethality did not follow a linear trend and appeared to reflect the consistently severe clinical profile of hospitalized patients, rather than external disruptions in health service delivery.

## Discussion

This ten-year retrospective study of TB-related hospitalizations in a tertiary public hospital in southeastern Brazil highlights the interplay between clinical severity, social vulnerability, and systemic diagnostic delays in a high-burden setting. Among 197 hospitalizations, most patients were young adult males (mean age: 41.8-years; 73.1%), of mixed race, with low educational attainment and high prevalence of substance use, malnutrition, and HIV coinfection. These demographic and clinical patterns reflect the persistent interaction of biological, social, and structural factors that continue to shape the burden and outcomes of TB in Brazil^2^, ^3^, ^4^, ^5^, ^6^, ^7^, ^19^, ^20^

The predominance of young adult males with low educational attainment and high levels of social vulnerability, including homelessness (9.1%) and substance use (64.0% smoking, 51.6% alcohol use, and 58.1% illicit drug use), is consistent with demographic patterns reported in other Brazilian and international cohorts^2^, ^3^, ^4^^,^^9^^,^^13^^,^^15^, ^16^, ^17^, ^18^ Notably, our study included transgender women (2.5%), a population frequently marginalized and disproportionately affected by poverty and HIV in Brazil^19^ Within this context of structural vulnerability, malnutrition (40.1%) emerged as a key predictor of in-hospital mortality (p = 0.011), corroborating global evidence that low BMI compromises immune responses and worsens tuberculosis prognosis^16^^,^^21^

HIV coinfection was found in 55.3% of patients, far exceeding Brazil’s national average of 11.4%,^2^ and 83.5% of coinfected individuals had CD4 counts ≤ 200 cells/mm^3^, with a median CD4 < 100 cells/mm^3^. Although HIV did not independently predict mortality in this cohort, this may be explained by overlapping risk factors and limited power. A large study in Italy^22^ similarly found that HIV coinfection (aOR = 3.4), CNS TB (aOR = 9.9), and miliary TB (aOR = 2.5) were all independently associated with mortality, paralleling our findings of severe forms and high immunosuppression.

Diabetes mellitus (6.6%) was the second most common comorbidity, comparable to reports in other middle-income settings^4^, ^23^, ^24^, ^25^ Although renal function was not systematically assessed, 3.0% had chronic kidney disease. In New Zealand, Kaur et al. (2023) showed that renal impairment in patients with TB-diabetes contributed substantially to hospitalizations and poor outcomes^18^ These findings underscore the need for integrated screening of metabolic and renal comorbidities in hospitalized TB patients. Notably, 18.2% of patients had no documented comorbidities. While this may reflect underreporting, it also raises the possibility of unrecognized risk factors or host susceptibilities.

Extrapulmonary (29.4%) and disseminated TB (21.8%) were frequent, and half of the extrapulmonary cases involved the central nervous system. This likely reflects referral bias due to the hospital’s neurology and neurosurgery services, but is also consistent with the increased prevalence of extrapulmonary disease among PLWHA^3^^,^^24^ Compared to a Portuguese nationwide study (Duarte et al., 2019), where pulmonary TB accounted for 66.4% of hospitalizations,^26^ our cohort presented a lower proportion of pulmonary TB (48.7%), suggesting more severe or diagnostically complex cases.

Diagnostic confirmation was achieved in 74.1% of cases, with AFB smear positivity in 37.8% and culture in 20%, similar to data from other tertiary centers in Brazil^4^^,^^24^^,^^25^ However, molecular testing (TRM-TB/Xpert MTB/RIF) was underused, performed in only 16% of patients, despite its high sensitivity and national availability. This aligns with findings from Chile, where molecular diagnostics were underutilized, and delays in diagnosis were common^27^ In our study, empirical treatment was initiated in 25.9% of cases, reflecting the diagnostic challenges in extrapulmonary and paucibacillary TB. Histopathology and ADA testing played a key role in these situations, although ADA’s sensitivity is variable depending on the anatomical site and clinical context^3^

A median symptom duration of 3.6-months prior to admission highlights a substantial diagnostic gap, exceeding delays reported in other high-burden settings^3^, ^4^, ^5^, ^9^, ^23^, ^27^ This delay helps explain why 74.1% of hospitalizations were primarily for diagnostic work-up, frequently triggered by acute complications such as respiratory failure (29.5%) or neurological symptoms^3^, ^9^, ^23^ Once admitted, the mean time to treatment initiation was 9-days, and the median length of hospital stay was 22-days. Compared with cohorts from lower-burden countries, this prolonged hospitalization reflects the complexity of managing advanced disease and the diagnostic challenges inherent to extrapulmonary and paucibacillary tuberculosis^9^^,^^24^, ^25^, ^26^, ^27^

The in-hospital mortality rate was 17.3%, higher than national estimates but consistent with tertiary care populations. Besides malnutrition, thrombocytopenia was also significantly associated with mortality (p = 0.022), consistent with data from the Philippines,^20^ which found platelet count as an independent predictor of death. Although neither HIV nor age were statistically significant predictors of death in our cohort, this may be due to sample size and interaction with other risk factors.

Contrary to expectations, we did not observe a sharp impact of the COVID-19 pandemic on TB-related admissions or mortality in our cohort. Admissions declined gradually from 2013 to 2021 and rose again in 2022. In contrast, Zaidi et al. (2023) reported a dramatic drop in TB diagnoses during the pandemic in Morocco, followed by a surge in severe extrapulmonary cases^28^

This study has several limitations. First, its retrospective design limits causal inference and the ability to assess temporal dynamics of symptom onset, diagnosis, and treatment response. Second, it was conducted in a single tertiary referral center, which may restrict generalizability to primary or regional settings. Third, critical social determinants, such as housing insecurity, incarceration, and substance use, may have been underreported. Fourth, the inclusion of clinically defined tuberculosis cases without microbiological confirmation may have introduced diagnostic heterogeneity, however, this approach reflects real-world clinical practice in high-burden tertiary settings, particularly in patients with extrapulmonary or paucibacillary disease, in whom microbiological confirmation is frequently not feasible. Fifth, post-discharge outcomes, including treatment completion, relapse, and long-term mortality, could not be assessed due to the lack of systematic outpatient follow-up data. Additionally, multivariable regression analysis was not performed due to the limited number of in-hospital deaths, which restricted adjustment for potential confounders such as age, HIV status, and comorbidities. Finally, the classification of tuberculosis forms and indications for hospitalization relied in part on clinician documentation, which may vary in accuracy and completeness in retrospective studies.

These findings highlight how the persistent intersection of diagnostic delays, severe clinical presentations, and structural social inequities continues to fuel tuberculosis-related hospitalizations and mortality in Brazil. Despite national advances in TB control, our data highlight persistent diagnostic gaps, underuse of molecular tools, and a high burden of comorbidities, particularly among marginalized populations. The substantial proportion of patients without confirmed microbiological diagnosis, prolonged symptom duration before admission, and frequent extrapulmonary involvement underscore the need for earlier detection and referral pathways. Addressing TB in this context demands more than biomedical solutions, it requires integrated public health strategies that incorporate social protection, comorbidity management, and health system strengthening. In high-burden urban centers, targeted interventions for vulnerable groups, including people living with HIV, the malnourished, and those experiencing homelessness, remain critical to reducing preventable hospitalizations and deaths.

## Ethics approval and consent to participate

The study was conducted in accordance with the Declaration of Helsinki, and approved by the Institutional Review Board of the Centro Universitário FMABC (CAAE: 69610323.4.0000.0082) and authorized by the HEMC clinical directorate.

## Informed consent statement

Patient consent was waived due to the design of study (retrospective, based on analyses of clinical and environmental samples).

## Consent for publication

All authors have read and agreed to the published version of the manuscript.

## Authors’ contributions

G.P.O.F.H: Conceptualization; Investigation; Writing-original draft; Data curation; G.B.P.: Writing-original draft; E.I.: Formal analysis; N.C.deA.D.: Data curation; Investigation; D.A.N.: Visualization; Writing-review and editing; O.H.M.L.: Supervision, conceptualization, review and editing; M.M.C.M.: Writing-original draft; Writing-review and editing; Supervision; Project administration. All authors have approved the final manuscript draft.

## Funding

The authors declare no funding.

## Data availability statement

The data that support the findings of this study are available from the corresponding author upon reasonable request.

## Conflicts of interest

None.

## References

[1] Global tuberculosis report 2023. Geneva: World Health Organization; 2023. Licence: CC BY-NC-SA 3.0 IGO.

[2] Ministério da Saúde (BR) (2025). Secretaria de vigilância em saúde e ambiente. departamento de hiv, aids, tuberculose, hepatites virais e infecções sexualmente transmissíveis. boletim epidemiológico tuberculose 2025. Brasília: ministério da saúde. https://www.gov.br/aids/pt-br/central-de-conteudo/boletins-epidemiologicos/2025/boletim-epidemiologico-tuberculose-2025/view.

[3] Ministério da Saúde (BR) (2019). Secretaria de vigilância em saúde. departamento de vigilância das doenças transmissíveis. manual de recomendações para o controle da tuberculose no Brasil. Brasília: Ministério da Saúde.

[4] É Zoé, L Furtado, Maria L., Rodrigues C., Monteiro A.S., de Oliveira AKN (2020). Perfil clínico e epidemiológico de pacientes com tuberculose diagnosticados em um hospital universitário. Rev Bras Pesq Saúde.

[5] Klautau G.B., Kuschnaroff TM. (2005). Clinical Forms and Outcome of Tuberculosis in HIV-Infected Patients in a Tertiary Hospital in São Paulo-Brazil. Brazilian J Infect Dis [Internet].

[6] dos Santos GM, AMM Carrijo, Paulinelli A.J.C., A Queiroz G de, e Silva L de LC, de Oliveira SV (2023 Mar 31). Hospitalizações por tuberculose na Região Sudeste: uma análise epidemiológica. Rev Med (Rio J).

[7] Ranzani O.T., Rodrigues L.C., Waldman E.A., Prina E., Carvalho CRR. (2018 Apr). Who are the patients with tuberculosis who are diagnosed in emergency facilities? An analysis of treatment outcomes in the state of São Paulo. Brazil. J Bras Pneumol..

[8] Banta J.E., Ani C., Bvute K.M., Lloren J.I.C., Darnell TA. (2020 Jan). Pulmonary vs. extra-pulmonary tuberculosis hospitalizations in the US [1998-2014]. J Infect Public Health.

[9] Ronald L.A., FitzGerald J.M., Benedetti A., Boivin J.F., Schwartzman K., Bartlett-Esquilant G., Menzies D. (2016 Nov 15). Predictors of hospitalization of tuberculosis patients in Montreal, Canada: a retrospective cohort study. BMC Infect Dis.

[10] Pinheiro N., Severo F., Queico C., Leite F., Capela M.V., Jacira Da M. (2007). Clinical and demographic characteristics of patients hospitalized with tuberculosis in Brasil between. J Bras Pneumol.

[11] Cortez A.O., de Melo A.C., Neves L., de O., Resende K.A., Camargos P. (2021). Tuberculosis in Brazil: one country, multiple realities. Jornal Brasileiro de Pneumologia.

[12] Arcêncio R.A., de Oliveira M.F., Villa TC. (2007). Internações por tuberculose pulmonar no Estado de São Paulo no ano de 2004 [Hospitalizations for pulmonary tuberculosis in the State of São Paulo in 2004]. Cien Saude Colet.

[13] Oliveira Junior A.R., Volpe-Chaves C.E., Lacerda MLGG, Bertucci A.A., Saad B.A.A., Oliveira C.T.F., Venturini J., Oliveira SMDVL, Paniago A.M.M. (2025 Feb 17). Factors associated with tuberculosis deaths during hospitalization in Midwest Brazil. Rev Inst Med Trop Sao Paulo.

[14] Beckwith P.G., Tlali M., Charalambous S., Churchyard G.J., Fielding K.L., Hoffmann C.J., Johnson S., Wood N., Grant A.D., Karat AS. (2021 Oct 18). Causes and outcomes of admission and investigation of tuberculosis in adults with advanced HIV in South African hospitals: data from the tb fast track trial. Am J Trop Med Hyg.

[15] Silva D.R., Muñoz-Torrico M., Duarte R., Galvão T., Bonini E.H., Arbex F.F. (2018 Mar 1). Risk factors for tuberculosis: diabetes, smoking, alcohol use, and the use of other drugs. Jornal Brasileiro de Pneumologia.

[16] Ockenga J., Fuhse K., Chatterjee S., Malykh R., Rippin H., Pirlich M. (2023 Apr 1). Tuberculosis and malnutrition: the european perspective. Clinical Nutrition.

[17] Aljohaney AA. (2018 Mar 1). Mortality of patients hospitalized for active tuberculosis in King Abdulaziz University Hospital, Jeddah, Saudi Arabia. Saudi Med J.

[18] Kaur R., Egli T., Paynter J., Murphy R., Perumal L., Lee A., Harrison A., Christmas T., Lewis C., Nisbet M. (2023 Sep). Tuberculosis and diabetes: increased hospitalisations and mortality associated with renal impairment. Intern Med J.

[19] Júnior S.F., Francisco PMSB, Nogueira PA. (2019 Aug 1). Knowledge, attitudes and practices regarding tuberculosis among transgender individuals in the city of São Paulo, Brazil. Ciencia e Saude Coletiva.

[20] Shimazaki T., Marte S.D., Saludar N.R.D., Dimaano E.M., Salva E.P., Ariyoshi K. (2013). Risk factors for death among hospitalised tuberculosis patients in poor urban areas in Manila, the Philippines. Int J Tubercul Lung Dis.

[21] Bhargava A., Chatterjee M., Jain Y., Chatterjee B., Kataria A., Bhargava M., Kataria R., D'Souza R., Jain R., Benedetti A., Pai M., Menzies D. (2013 Oct 24). Nutritional status of adult patients with pulmonary tuberculosis in rural central India and its association with mortality. PLoS One.

[22] Pipitò L., Colomba C., Mancuso A., Catania B., Cuccia A., Sergio M., Iaria C., Cascio A. (2023 Sep). Hospitalizations for tuberculosis in Sicily over the years 2009-2021: Clinical features, comorbidities, and predictors of mortality. J Infect Public Health.

[23] Silva D.R., Silva L.P., Dalcin PTR. (2014). Tuberculosis in hospitalized patients: clinical characteristics of patients receiving treatment within the first 24h after admission. J Bras Pneumol.

[24] Silva D.R., Menegotto D.M., Schulz L.F., Gazzana M.B., Dalcin PDTR. (2010 Feb). Factors associated with mortality in hospitalized patients with newly diagnosed tuberculosis. Lung.

[25] Cristina M., Perrechi T., Ribeiro SA. (2011). Desfechos de tratamento de tuberculose em pacientes hospitalizados e não hospitalizados no município de São Paulo. J Bras Pneumol.

[26] Duarte F.F., Santos J., Duarte R., Freitas A. (2019 Feb). Burden of tuberculosis hospitalizations in Portugal From 2000 to 2015. Arch Bronco (Engl Ed).

[27] Fica A., Osorio C., Muñoz C., Olivares F., Wenger R., Navarrete M., Aravena M., Carrasco C., Toro N., Silva R. (2023 Jun). Admissions by tuberculosis in a regional reference center. a complex and worrying scenario. Rev Med Chil.

[28] Zaidi S., Errami A., Belkhou I., Elkhaldi M., Ailal F., Benhsaien I., Adnane F., Amenzoui N., Abkari A., Bousfiha AA. (2024 Jul 5). Impact of COVID-19 on child tuberculosis hospitalization. Tunis Med.

